# Systemic Inflammation (C-Reactive Protein) in Older Chinese Adults Is Associated with Long-Term Exposure to Ambient Air Pollution

**DOI:** 10.3390/ijerph18063258

**Published:** 2021-03-22

**Authors:** Mona Elbarbary, Artem Oganesyan, Trenton Honda, Geoffrey Morgan, Yuming Guo, Yanfei Guo, Joel Negin

**Affiliations:** 1 Faculty of Medicine and Health, Sydney School of Public Health, The University of Sydney, Sydney, NSW 2006, Australia; Geoffrey.morgan@sydney.edu.au (G.M.); joel.negin@sydney.edu.au (J.N.); 2 Department of Hematology and Transfusion Medicine, National Institute of Health, Yerevan 0051, Armenia; a.t.oganesyan@gmail.com; 3 Bouvé College of Health Sciences, Northeastern University, Boston, MA 02115, USA; t.honda@northeastern.edu; 4 School of Public Health, University Centre for Rural Health, The University of Sydney, Sydney, NSW 2006, Australia; 5 Department of Epidemiology and Preventive Medicine, School of Public Health and Preventive Medicine, Monash University, Clayton, VIC 3800, Australia; Yuming.Guo@monash.edu; 6 Shanghai Municipal Center for Disease Control and Prevention, Shanghai 200336, China; guoyanfei@scdc.sh.cn

**Keywords:** air pollution, C-reactive protein, inflammatory marker, CVD risk, China, elderly

## Abstract

There is an established association between air pollution and cardiovascular disease (CVD), which is likely to be mediated by systemic inflammation. The present study evaluated links between long-term exposure to ambient air pollution and high-sensitivity C reactive protein (hs-CRP) in an older Chinese adult cohort (*n* = 7915) enrolled in the World Health Organization (WHO) study on global aging and adult health (SAGE) China Wave 1 in 2008–2010. Multilevel linear and logistic regression models were used to assess the associations of particulate matter (PM) and nitrogen dioxide (NO_2_) on log-transformed hs-CRP levels and odds ratios of CVD risk derived from CRP levels adjusted for confounders. A satellite-based spatial statistical model was applied to estimate the average community exposure to outdoor air pollutants (PM with an aerodynamic diameter of 10 μm or less (PM_10_), 2.5 μm or less (PM_2.5_), and 1 μm or less (PM_1_) and NO_2_) for each participant of the study. hs-CRP levels were drawn from dried blood spots of each participant. Each 10 μg/m^3^ increment in PM_10_, PM_2.5_, PM_1_, and NO_2_ was associated with 12.8% (95% confidence interval; (CI): 9.1, 16.6), 15.7% (95% CI: 10.9, 20.8), 10.2% (95% CI: 7.3, 13.2), and 11.8% (95% CI: 7.9, 15.8) higher serum levels of hs-CRP, respectively. Our findings suggest that air pollution may be an important factor in increasing systemic inflammation in older Chinese adults.

## 1. Introduction

Air pollution is a significant global health challenge [1]. According to recent data from the World Health Organization (WHO), 91% of the world’s population lives in places where air pollution exceeds the WHO recommendations [2], contributing to over four million premature deaths every year, as well as the development and exacerbation of numerous chronic diseases, such as cardiovascular disease (CVD), respiratory disorders, and cancer [3,4].

Among air pollutants, the negative impact on health is best established for particulate matter of less than 10 and 2.5 microns in diameter (PM_10_ and PM_2.5_, respectively) [5,6,7]. Outdoor PM_2.5_ was shown to be the fifth leading cause of mortality in 2015 [8], and the worst impacts from ambient air pollution are seen in low- and middle-income countries due to rapid industrialization and a lack of environmental regulation [8]. Southeast Asia is at the highest risk in this regard [2]. Moreover, populations from these geographic areas bear a high burden of chronic, noncommunicable diseases such as CVD [9]. The impact of air pollution on the development and progression of CVD is of particular concern, as numerous studies have demonstrated important and significant associations consistent with a causal relationship between air pollution and CVD [10,11,12]. CVD accounts for most of the mortality attributed to air pollution. Conversely, ambient air pollution is attributed to 17.1% and 14.2% of deaths from ischemic heart disease and cerebrovascular disease, respectively [8].

One of the primary suggested mechanisms via which outdoor air pollutants might lead to CVD is chronic systemic inflammation [13]. Air pollution can directly lead to pulmonary inflammation through activation of alveolar macrophages and the upregulation of inflammatory cytokine expression, such as tumor necrosis factors and interleukins [14,15]. These inflammatory cytokines can, in sufficient concentration, lead to a hepatic acute phase response, with resultant increases in numerous serum proteins associated with systemic inflammation, including C-reactive protein (CRP). CRP is a well-known inflammatory biomarker and can be a valuable indicator for both acute and chronic inflammation [16]. Although high blood levels of CRP can be caused by a range of health conditions, chronic and sustained increases in this protein have been consistently tied to the development and progression of CVD [17].

Several previous studies studied the association between particulate matter air pollution and CRP; however, significant questions remain [18,19,20]. For example, in a recent meta-analysis of 40 observational studies, including a total of 244,681 participants, investigators found that, while long-term exposure to ambient air pollution was more strongly associated with CRP levels than short-term exposure, the majority of prior literature focused on short-term exposure windows [21]. Additionally, while the burden of air pollution exposure is known to be significantly greater in developing nations, the studies linking PM to CRP so far have been disproportionally performed in populations from economically developed regions [22], such as Europe and the United States (US) [21]. To date, there have been no studies evaluating the long-term relationship between ambient air pollution and levels of CRP in the Chinese population, which is still, despite substantial progress, exposed to high levels of outdoor pollution [23]. The results of studies assessing associations between NO_2_ exposure and CRP levels were inconclusive [24,25,26,27], and no data have been published regarding PM_1_, which is believed to be even more toxic than other pollutants [28]. Lastly, most prior studies were conducted on young populations, which may be at lower risk for the detrimental cardiovascular consequences of air pollution-induced chronic inflammation [21].

Thus, the current study aims to fill these gaps in the literature by examining the association between exposure to long-term, ambient air pollutants and levels of serum hs-CRP among older Chinese adults.

## 2. Materials and Methods

### 2.1. Study Popultation

The current study is based on the data collected from the WHO’s study on global aging and adult health (SAGE)—a longitudinal study evaluating in detail the health and wellbeing of adult populations in middle-income countries (China, Ghana, Mexico, India, Russia, and South Africa). Our study analyzed the cross-sectional, baseline, interview-based survey data of older Chinese adult respondents from 2008 to 2010 (SAGE China Wave 1). Multistage clustering and probability sampling were applied for participant recruitment, described in detail elsewhere [29]. This resulted in a nationally representative sample from 64 townships across China. The study was approved by the Ethics Committee of The Chinese Center for Disease Control and Prevention.

### 2.2. Exposure Assessment

Predictive models estimated exposure concentrations of PM_1_, PM_2.5_, PM_10_, and NO_2_ on the basis of satellite remote sensing, meteorology, land use, and other data combined with ground-monitored information on the pollutants from stations throughout mainland China. For PM_1_, daily ground-level measurements were acquired from 77 stations of the China Atmosphere Watch Network (CAWNET) during September 2013 and December 2014. For the other three pollutants, the data were obtained from 1479 stations of the China National Environmental Monitoring Center (CNEMC) from May 2014 to December 2016.

Detailed descriptions of the predictive models for each pollutant can be found elsewhere [30,31]. In short, two National Aeronautics and Space Administration (NASA) Moderate Resolution Imaging Spectroradiometer (MODIS) data processing algorithms and inverse variance weighting at 0.1° (10 km) grid cell resolution provided data on aerosol optical depth (AOD). These data, along with data on land use, vegetation, and meteorology, were combined with ground-monitored PM_1_ data via a generalized additive model for the prediction of daily PM_1_ grid cell concentrations from 2005 to 2014. The same methods were applied for the prediction of grid cell concentrations of PM_2.5_ and PM_10_ from 2005 to 2016.

For NO_2_, the OMI-NO_2_ level 3 data product (OMNO_2_d version 3) provided data on satellite-derived tropospheric column densities of NO_2_ (molecules/cm^2^) at 0.25° (13 × 24 km^2^) resolution [32]. Predictions of daily grid cell concentrations for NO_2_ from 2013 to 2016 were obtained via a random forest model using ground-monitored NO_2_ data linked with data on satellite NO_2_, vegetation, land use, road density, and meteorology [33].

Monitored pollutant data and 10-fold cross-validation were used to assess the predictive ability of the models for all four pollutants (Table S2, Supplementary Materials). The long-term exposure to air pollutant was defined as the moving average concentrations in the participants’ township for the 1, 3, and 5 year periods prior to the participant entering the study. As each township entered the study and provided biological samples at a different time during the 2008–2010 baseline study period, the moving average exposure was different for each township. Participants’ community locations were geo-coded, and participant-specific long-term concentration estimates were calculated for PM_10_, PM_2.5_, PM_1_, and NO_2_.

### 2.3. Hs-CRP Level Measuremenets

Hs-CRP is a systemic inflammatory marker that is produced in the liver after stimulation through cytokines. Hs-CRP levels were measured using the dried blood spot (DBS) technique considering difficulties related to the collection, processing, and storage of the serum specimens. DBS was validated as a feasible tool for the assessment of CRP levels on a population level, as its values have been consistently correlated with those measured by standard serum sampling [34,35]. Hs-CRP levels were analyzed using an enzyme-linked immunosorbent assay (ELISA) (Diagnostic Biochem Canada Inc, London, Canada) with less than 16% of the coefficient of variability (CV) for the assay. A higher accuracy of the detection range (0.01–10 mg/L) of hs-CRP is most applicable for the establishment of low-degree inflammatory states, especially in individuals without clinical manifestations of inflammation and CVD [36].

### 2.4. Covariates

Covariates were selected for inclusion in our health effects models according to prior associations with air pollution and hs-CRP. These include demographic, health behavior, socioeconomic status, indoor air pollution, and comorbid disease variables. Demographic data included sex, age, and body mass index (BMI). Health behavior variables included dietary intake of fruits and vegetables, alcohol and tobacco use, and physical activity. Fruit and vegetable consumption was included in a binary fashion as either sufficient (two and more daily servings of fruits and three or more daily servings of vegetables) or insufficient. Alcohol use (current use or no current use) and smoking status (current use or no current use) were likewise included as binary variables. Physical activity was characterized by the global physical activity questionnaire, which included questions on the duration, frequency, and intensity of physical activity during work, transport activities, and recreation/leisure time activities. Correspondingly, these responses were collapsed into three groups (low, moderate, and high physical activity) defined according to total energy requirements in metabolic equivalents representing the intensity and time spent on each activity. Socioeconomic variables included the level of education and self-reported household income. The level of education was categorized as having attended (1) no school, (2) primary school, (3) middle school, or (4) high school and higher. Self-reported household income was included in a binary fashion (high versus low), using the median household income of 20,000 CNY as a cutoff. Indoor pollution was assessed using data on the type of fuel being used for cooking at home. It was, thus, classified as either clean (electricity and natural gas) or unclean (coal, wood, dung, and agricultural residues). Lastly, self-reported comorbidities, such as hypertension, chronic lung disease, and diabetes, were also assessed and included through participant self-report.

### 2.5. Statistical Analysis

We examined the association between log hs-CRP levels and 10 μg/m^3^ increases in the 3 year moving average of annual average PM_10_, PM_2.5_, PM_1_, and NO_2_ in single-pollutant, multilevel linear, and logistic regression models, where participants were considered as the first-level unit and the township as the second-level unit. For linear models, hs-CRP was log-transformed to approximate a more normal distribution; for logistic regression models, we examined the probability of a hs-CRP >3 mg/L, as this cutoff point has been used as a clinical indicator for high-risk CVD [37]. In all models, we included the following covariates: age, sex, BMI, tobacco use, physical activity, education level, fruit and vegetable intake, alcohol use, type of fuel used at home, median household income, and location of residence (urban/rural).

Stratified analyses were performed to investigate possible effect modification. Stratifications were made for several socioeconomic values that could have a direct impact on susceptibility to inflammation or could be linked with pollution [38,39]. These values involved level of education (high-school graduation or less versus college degree or more) and annual household income (<20,000 CNY versus ≥20,000 CNY with the median used as the cutoff point). Further subdivisions included several health indicators, conditions, or behaviors with a potential to enhance inflammation, such as age (<65 versus >65 years), diabetes, and chronic lung disease (using self-reported diagnosis). The evaluation of effect modification was carried out by the inclusion of multiplicative terms between pollutant variables and the potential effect modifiers in the adjusted models. For the significance of effect modification, the *p*-value for the hypothesis test of the interaction was selected as <0.01. The percentage change in hs-CRP was used to present the results of the linear regression analysis and the percentage change in probability of hs-CRP above 3 mg/L (CVD risk threshold) was used for the results of the binary model. Both results were calculated using [exp(10 × β) − 1] × 100.

Several sensitivity analyses were carried out to ensure our results were robust to different model specifications. These included (1) examining different pollution exposure windows (i.e., 1 year and 5 year), and (2) excluding participants with comorbidities which may represent causal intermediates between air pollution exposures and CRP levels, such as respiratory and cardiovascular comorbidities. For all analyses, STATA version 15 (StataCorp, College Station, TX, USA) was used. The statistical significance was determined as a *p*-value <0.05.

## 3. Results

### 3.1. Study Population

The baseline characteristics of participants involved in this study are presented in Table 1. In total, 7915 individuals were included in the final analysis, representing 59.2% of the study population of 13,367 aged 50 and older. Baseline demographic characteristics for the participants with complete hs-CRP measurements and nonparticipants due to incomplete hs-CRP were compared (Table S2, Supplementary Materials).

The mean age of the participants was 63.22 years (±9.35), 52% of whom were female. Most of the participants were residents of rural areas (54%) who used clean types of fuel at home (58%) with no current use of tobacco (67%) or alcohol (70%) and who had sufficient intake of fruits and vegetables (58%). About 53% reported having less than 20,000 CNY of annual household income. Most of the study population had no history of diabetes (93%) or chronic lung disease (92%).

### 3.2. Air Pollution Exposure

**Figure 1:**
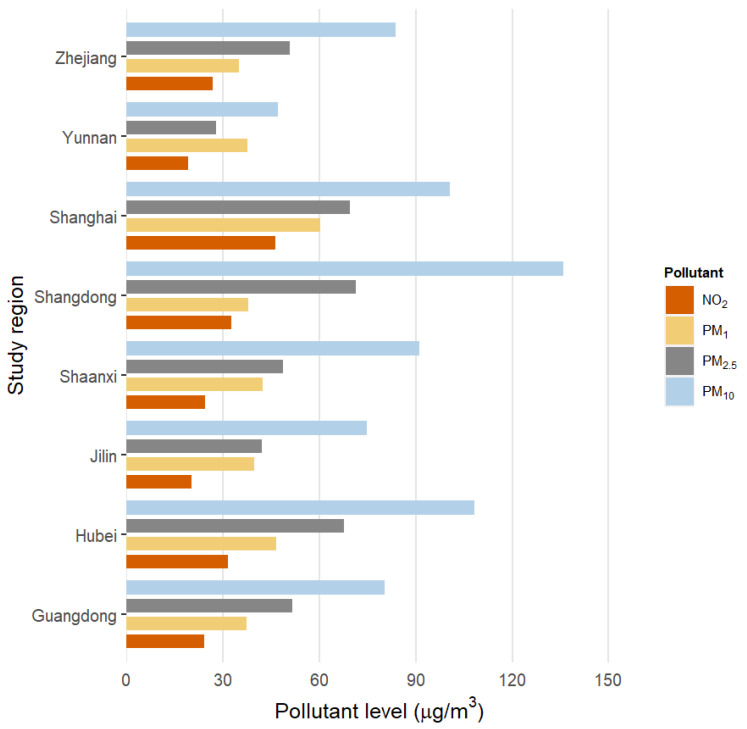
Distributions of 3-year average concentrations of air pollutants in study on global aging and adult health (SAGE) China study regions.

Figure 1 shows the distribution of residential ambient air pollution concentrations. Mean (±SD) annual estimates of PM_10_, PM_2.5_, and NO_2_ were 91.11 (±28.95 µg/m^3^), 54.02 (±17.02 µg/m^3^), and 28.97 (±22.42 µg/m^3^), respectively. NO_2_ concentrations were highly correlated with PM_2.5_ (*r* = 0.92), but less so with PM_10_.

### 3.3. Association of Exposure to PM and NO_2_ with hs-CRP Levels

**Figure 2:**
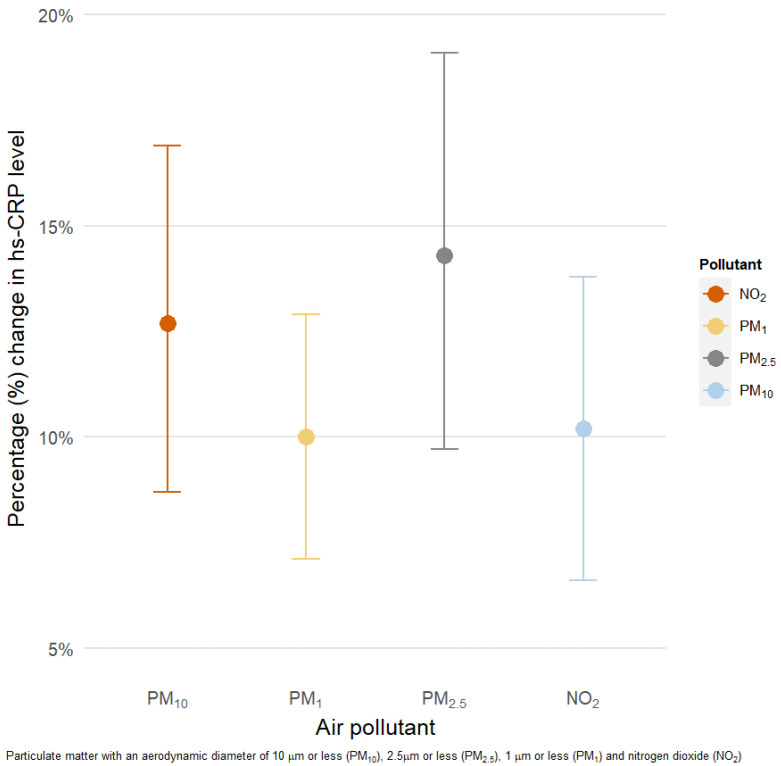
Percentage change (95% confidence interval (CI)) in high-sensitivity C reactive protein (hs-CRP) levels associated with 10 μg/m^3^ increase in 3 year moving averages of air pollution. PM_10_ = particulate matter with a diameter of 10 μm or less, PM_2.5_ = with a diameter of 2.5 μm or less, PM_1_ = particulate matter with a diameter of 1 μm or less, NO_2_ = nitrogen dioxide. Models were adjusted for age, sex, BMI, tobacco use, physical activity, education level, fruit and vegetable intake, alcohol use, type of fuel used at home, median household income, and location of residence (urban/rural).

For all pollutants, we found statistically significant positive associations with serum hs-CRP (Figure 2). Each 10 μg/m^3^ increment in 3 year moving averages of PM_10_, PM_2.5_, PM_1_, and NO_2_ was associated with 12.8% (95% confidence interval (CI): 9.1, 16.6), 15.7% (95% CI: 10.9, 20.8), 10.2% (95% CI: 7.3, 13.2), and 11.8% (95% CI: 7.9, 15.8) higher serum levels of hs-CRP, respectively.

### 3.4. Odds Ratio of CVD Risk Increase

**Figure 3:**
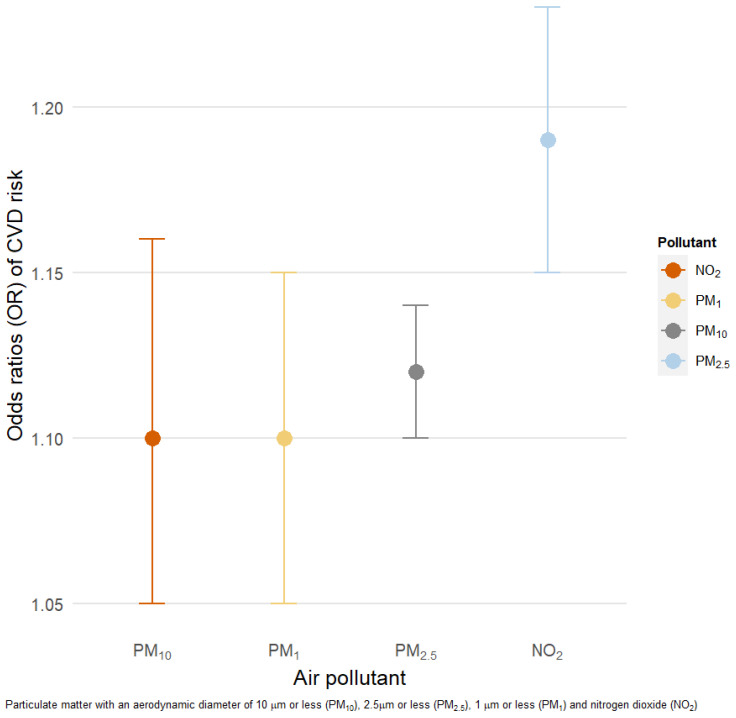
Association between a 10 μg/m^3^ increment in 3 year moving averages of air pollution and CVD risk. Models adjusted for age, sex, BMI, tobacco use, physical activity, education level, fruit and vegetable intake, alcohol use, type of fuel used at home, median household income, and location of residence (urban/rural). OR = odds ratio.

Figure 3 presents the results of the logistic regression models. We observed higher odds ratios (ORs) of CVD risk (defined as hs-CRP > 3 mg/L) for 10 μg/m^3^ increments of PM_10_ (OR: 1.12 (95% CI: 1.10, 1.14)), PM_2.5_ (1.19 (95% CI: 1.15, 1.23)), PM_1_ (1.10 (95% CI: 1.05, 1.15)), and NO_2_ (1.10 (95% CI: 1.05, 1.16)).

### 3.5. Sensitivity Analysis

Our sensitivity analyses examining 5 year and 1 year exposure windows (Figures S1 and S2, Supplementary Materials) did not differ meaningfully from our main models, in terms of both the magnitude and the direction of the effect estimates, for every pollutant assessed. Likewise, when excluding potential causal intermediates (respiratory and cardiovascular comorbidities) we did not observe important changes in our effect estimates (data not shown).

### 3.6. Effect Modification

The results of our effect modification models are presented in Table 2 for linear models and in Table 3 for logistic regression models. In our linear models, we observed a statistically significant effect modification for median household income on PM_1_ exposure (*p* < 0.001), with larger associations observed for household incomes above the median (percentage change hs-CRP: 8.38, 95% CI: 3.46, 13.53) than below the median (% change hs-CRP: 7.56, 95% CI: 3.62, 11.64). Similar effects for NO_2_, PM_2.5_, and PM_10_ did not reach statistical significance. Participants over 65 years of age had nominally, although nonsignificant, larger effects due to PM_2.5_ and PM_10_ exposure. No significant effect modification was observed for other investigated variables in our linear models. A similar pattern was observed in our logistic regression models, although no statistically significant effect modification was observed (Table 3).

## 4. Discussion

We found that NO_2_ and multiple size fractions of PM were all strongly and significantly associated with increased levels of hs-CRP in an older Chinese population. The significance of these associations persisted after several sensitivity and subgroup analyses. All investigated air pollutants were strongly and significantly associated with clinical important elevations in hs-CRP (defined as hs- CRP > 3 mg/L), a level which has been previously associated with the development of CVD.

Our results are consistent with findings from most previous studies of PM, although the prior literature was mainly limited to short-term investigations in high-income countries demonstrating larger increases in CRP levels compared to those presented here [21]. This can presumably be explained by the substantially larger cumulative effects on tissue damage and inflammation which occur with long-term exposure to elevated air pollution levels [21].

A pooled data meta-analysis of nine studies by Liu et al. demonstrated statistically significant associations between elevations in PM and increases in serum CRP levels (*p* = 0.003), indicating an 18.01% and 5.61% increase in CRP with every 10 μg/m^3^ increment in PM_2.5_ and PM_10_, respectively [21]. Moreover, after stratification by study location, i.e., Asian populations (both from Taiwan), the effects remained strong (*p* < 0.001). Several publications not included in this systematic review also supported these findings [19,40,41]. Nonetheless, a study by Tsai et al. did not find any significant association with PM_10_ exposure in a Swiss cohort of 8121 participants [42].

The present study is among the first to evaluate the negative associations between PM_1_ and inflammation. PM_1_ is rarely assessed in health studies compared to other air pollutants. As a result, there is no standardized reference for PM_1_ levels set by the WHO. PM_1_ may play a greater role than PM_2.5_ in associations with CVD [43]. Moreover, fine and ultrafine particulates (with a diameter of <2.5 μm and <1 μm, respectively) are even more harmful since they can penetrate deeper into lung tissues and spread throughout the body via the bloodstream, causing both acute and chronic health effects [28,44,45]. Consistent with this, increased exposure to PM has been shown to be associated with an increased prevalence of chronic lung diseases, respiratory infections, CVD, and diabetes, whereby inflammation is regarded as the key underlying mechanism of adverse effects from air pollution [46].

These associations, however, have been less consistent in the case of nitrogen dioxide. Multiple studies from European countries, as well as one study from the US and another one from Taiwan, failed to demonstrate any substantial links between levels of ambient NO_2_ and serum CRP levels [24,26,27,40,47,48,49,50,51,52]. On the contrary, an analysis of two large European cohorts involving 51,459 participants found a 1.9% increase in hs-CRP in association with a 7.4 μg/m^3^ increment in NO_2_ exposure [53]. Our study revealed stronger associations (11.8% per 10 μg/m^3^ increase in 3 year moving averages of outdoor NO_2_), which can be possibly explained by the older mean age of participants (63.2 vs. 47.6 years), as well as greater co-exposure of other pollutants.

Contrary to some of the previous research, our study did not find any effect modification from common covariates, such as age, sex, smoking, and income level. The only exception was that the individuals with an average household income of more than 20,000 CNY had a higher percentage change in hs-CRP levels with every 10 μg/m^3^ exposure to PM_1_. Prior evidence showed stronger associations among the elderly, smokers, people with diabetes and higher BMI, alcohol consumers, those with poorer education, and those with lower income levels. Use of certain medications, such as hormone therapy and statins, as well as marital status, was also shown to have an effect modification [54,55,56].

To our best knowledge, this is the first study to investigate the long-term effects of air pollution exposure and levels of hs-CRP among residents from China. Previous studies were primarily focused on populations from European and North American countries, apart from two reports from Taiwan [49,57]. In an analysis of 30,034 Taiwanese residents, Zhang et al. showed that every 5 μg/m^3^ of PM_2.5_ increase was linked with an average of 1.31% higher concentrations of CRP [57], considerably lower than the effects observed in our study (15.7%, 95% CI: 10.9, 20.8), which are more comparable with those from European and US cohorts [18,19,41,54,55,58]. On the contrary, Huang et al. found no statistically significant associations between either PM or NO_2_ levels and increases in serum CRP in a small sample of 175 patients undergoing continuous peritoneal dialysis [49]. In general, the magnitude of positive associations between CRP and PM_10_ seen in our participants was similar to those seen in previous studies [18,40,53,55].

Countries with developing economies and booming industrialization suffer most from environmental problems, including air pollution [22]. For example, China, despite the progress made over recent years, still has one of the highest levels of outdoor air pollution compared to the rest of the world [23,59]. China also shares one of the highest burdens from CVDs, to which 40% of all deaths in the country are attributed [60].

The elderly population may be particularly susceptible to the negative cardiovascular impacts from ambient air pollution, given their substantial burden of chronic inflammatory disorders, perturbations in immune function, and changes to physical activity that accompany aging [12,61,62]. With the global population projected to get older over the next decades, the combination of elevated exposure and susceptibility may lead to significant increases in disease burden associated with air pollution exposure [63,64,65].

### Strength and Limitations

The findings of the current study are enhanced by a number of significant methodological strengths. First, we were able to investigate the detrimental health effects of air pollution in a specific population known to be at particular risk: older adults from a developing country with higher-than-average air pollution. Second, the study sample included a geographically diverse population of residents which is representative of China. Third, individual-level data on many common risk factors enabled adjustment for a range of personal confounders. Fourth, the applied models incorporated satellite-based estimates of PM exposures, which, despite the absence of air monitoring data, created total spatial coverage among participants of this study. Lastly, the present work is among very few studies examining associations between CRP and PM_1_ and NO_2_, the data on which are largely limited and inconsistent. The findings of this study can help to fill the gap in PM_1_ data, further serving as an evidence background for pollution standards, public policies, and guidelines for concentration cut-offs.

Our study also has limitations. The significance of the results is limited by its cross-sectional design, restricting the observed associations to a single time point, although estimates for air pollution exposure were derived from 2005–2007 with hs-CRP concentrations measured in 2008–2010 as outcomes. Data on other important personal confounders of participants, which potentially could have impacted the levels of hs-CRP, such as the use of anti-inflammatory medications, second-hand smoke exposure, and presence of chronic or acute inflammatory conditions, were unfortunately not obtained during the survey. Additionally, no information on specific chemical components of PM, which could have determined the effects, was available. Furthermore, the findings should be taken cautiously since the inflammatory response was characterized by only one biomarker, which has its own limitations in terms of sensitivity and specificity. Our models did not include detailed information about residential differences, such as socioeconomic status, healthcare access, available green space, or temperature changes, all of which are deemed to be potential confounders for the exposure and outcome. The likelihood of exposure misclassification is also increased by the absence of the participants’ specific activity patterns, such as traffic and indoor time. Although we were not able to adjust for short-term air pollution exposure, which may potentially affect the long-term associations between inflammatory markers and outdoor air pollution exposure, measurements of hs-CRP excluded participants with CRP levels >10 mg/L, which reduced the likelihood of impact on outcome measurements, as previous long-term investigations also demonstrated no effect of short-term pollution on long-term associations [18,26,55]. Lastly, not all surveyed participants consented to provide their blood samples, which limited the generalizability of our findings and may have created a selection bias, as the final sample consisted of healthier, wealthier, and more educated participants. Nonetheless, the strength of observed associations is supported by statistical robustness, with the PR and CI being well above the value of one.

## 5. Conclusions

In our study in a nationally representative sample of older Chinese adults, we observed significant and consistent associations between long-term concentrations of ambient air pollution (PM_10_, PM_1_, and NO_2_) and high-sensitivity C reactive protein (hs-CRP). Our findings add further to the literature suggesting that air pollution may be an important factor in increasing systemic inflammation in older Chinese adults.

## Figures and Tables

**Table 1:** Baseline characteristics of study participants.

Characteristics	Mean	SD or %
PM_10_ 1 year (μg/m^3^)	90.23	28.80
PM_2.5_ 1 year (μg/m^3^)	53.94	17.08
PM_1_ 1 year (μg/m^3^)	43.67	13.04
NO_2_ 1 year (μg/m^3^)	30.52	12.36
Age (years)	63.22	9.35
BMI (kg/m^2^)	24.11	4.81
Systolic blood pressure (mmHg)	148.26	24.55
Diastolic blood pressure (mmHg)	84.64	13.55
Total annual household incomes		
• ≤20,000 CNY	4181	52.82
• >20,000 CNY	3512	44.37
Sex (*n*, %)		
• Male	3774	47.68
• Female	4141	52.32
Smoking status		
• Current tobacco use	2615	33.14
• No current tobacco use	5275	66.86
Alcohol use		
• Current alcohol drinking	2377	30.19
• No current alcohol drinker	5496	69.81
Education		
• No formal education	1762	22.26
• Primary school	3173	40.09
• Middle school	1521	19.22
• High school or higher	1459	18.43
Place of residence		
• Rural	4276	54.02
• Urban	3639	45.98
Physical activity		
• Low level	2707	34.31
• Moderate level	2243	28.43
• High level	2939	37.25
Nutrition		
• Insufficient intake of fruits and vegetables	3336	42.15
• Sufficient intake of fruits and vegetables	4579	57.85
Type of fuel used at home		
• Clean	4588	58.42
• Unclean	3265	41.58
History of Diabetes		
• Yes	554	7.06
• No	7298	92.94
History of Chronic lung diseases		
• Yes	629	8
• No	7236	92

BMI = body mass index, PM_10_ = particulate matter with a diameter of 10 μm or less, PM_2.5_ = with a diameter of 2.5 μm or less, PM_1_ = particulate matter with a diameter of 1 μm or less, NO_2_ = nitrogen dioxide.

**Table 2:** Stratified analysis of percentage change in hs-CRP level with 10 μg/m^3^ increase in 3 year moving averages in each pollutant level.

Characteristics	PM_10_ % (95% CI)	PM_2.5_ % (95% CI)	PM_1_ % (95% CI)	NO_2_ % (95% CI)
Sex	*p* = 0.09	*p* = 0.26	*p* = 0.25	*p* = 0.50
• Male	11.21 ^a^ (6.44, 16.18)	13.73 (7.21, 20.64)	9.76 (5.68, 14.01)	10.16 (4.86, 15.73)
• Female	12.58 ^a^ (7.65, 17.75)	16.06 (9.53, 22.98)	9.71 (5.73, 13.85)	11.91 (96.56, 17.52)
Smoking	*p* = 0.11	*p* = 0.34	*p* = 0.35	*p* = 0.52
• Yes	7.22 ^b^ (2.2, 12.67)	7.81 (0.53, 15.63)	6.56 (1.86, 11.49)	3.53 (−2.48, 9.92)
• No	14.29 ^b^ (9.75, 19.01)	17.95 (11.99, 24.24)	10.76 (7.18, 14.46)	14.07 (9.29, 19.06)
Age	*p* = 0.02	*p* = 0.03	*p* = 0.68	*p* = 0.34
• ≤65 years	11.34 ^c^ (6.93, 15.93)	13.41 (7.59, 19.54)	10.67 (7.13, 14.33)	11.69 (6.97, 16.63)
• >65 years	13.78 ^c^ (8.07, 19.80)	19.22 (11.26, 27.75)	9.89 (4.93, 15.08)	11.70 (5.25, 18.55)
Income	*p* = 0.08	*p* = 0.49	*p* < 0.001	*p* = 0.06
• ≤20,000 CNY	9.57 ^d^ (4.58, 14,80)	10.35 (3.47, 17.68)	7.56 (3.62, 11.64)	5.46 (0.20, 10.99)
• >20,000 CNY	6.33 ^d^ (0.59, 12.39)	9.55 (1.34, 18.42)	8.38 (3.46, 13.53)	9.19 (2.37, 16.49)

PM_10_ = particulate matter with a diameter of 10 μm or less, PM_2.5_ = with a diameter of 2.5 μm or less, PM_1_ = particulate matter with a diameter of 1 μm or less, NO_2_ = nitrogen dioxide. ^a^ Adjusted for age, BMI, tobacco use, physical activity, education level, fruit and vegetable intake, alcohol use, type of fuel used home, household annual income, location of residence (urban/rural). ^b^ Adjusted for age, sex, BMI, physical activity, education level, fruit and vegetable intake, alcohol use, type of fuel used at home, household annual income, and location of residence (urban/rural). ^c^ Adjusted for sex, BMI, tobacco use, physical activity, education level, fruit and vegetable intake, alcohol use, type of fuel used at home, household annual income, and location of residence (urban/rural). ^d^ Adjusted for age, sex, BMI, tobacco use, physical activity, education level, fruit and vegetable intake, alcohol use, type of fuel used at home, and location of residence (urban/rural).

**Table 3:** Stratified analysis of the probability of increased risk of CVD associated with 10 μg/m^3^ increase in 3 year moving averages in each pollutant level.

Characteristics	PM_10_ OR (95% CI)	PM_2.5_ OR (95% CI)	PM_1_ OR (95% CI)	NO_2_ OR (95% CI)
Sex	*p* = 0.93	*p* = 0.66	*p* = 0.07	*p* = 0.309
• Male	1.11 (1.08, 1.15) ^a^	1.19 (1.12, 1.26) ^a^	1.07 (0.99, 1.15) ^a^	1.11 (1.03, 1.20) ^a^
• Female	1.10 (1.08, 1.13) ^a^	1.20 (1.15, 1.26) ^a^	1.11 (1.04, 1.19) ^a^	1.15 (1.07, 1.23) ^a^
Smoking	*p* = 0.78	*p* = 0.58	*p* = 0.06	*p* = 0.051
• Yes	1.12 (1.07, 1.16) ^b^	1.17 (1.09, 1.25) ^b^	1.00 (0.92, 1.10) ^b^	1.03 (0.93, 1.13) ^b^
• No	1.10 (1.08, 1.13) ^b^	1.20 (1.16, 1.25) ^b^	1.13 (1.07, 1.21) ^b^	1.18 (1.11, 1.26) ^b^
Age	*p* = 0.06	*p* = 0.04	*p* = 0.35	*p* = 0.08
• ≤65 years	1.08 (1.06, 1.11) ^c^	1.14 (1.08, 1.20) ^c^	1.08 (1.01, 1.15) ^c^	1.05 (0.98, 1.13) ^c^
• >65 years	1.13 (1.11, 1.16) ^c^	1.27 (1.20, 1.33) ^c^	1.13 (1.04, 1.21) ^c^	1.25 (1.16, 1.36) ^c^
Income	*p* = 0.73	*p* = 0.93	*p* = 0.05	*p* = 0.86
• ≤20,000 CNY	1.10 (1.07, 1.13) ^d^	1.15 (1.09, 1.22) ^d^	1.02 (0.95, 1.09) ^d^	1.08 (0.99, 1.18) ^d^
• >20,000 CNY	1.10 (1.07, 1.12) ^d^	1.18 (1.13, 1.24)	1.08 (1.00, 1.17) ^d^	1.12 (1.05, 1.20) ^d^

PM_10_ = particulate matter with a diameter of 10 μm or less, PM_2.5_ = with a diameter of 2.5 μm or less, PM_1_ = particulate matter with a diameter of 1 μm or less, NO_2_ = nitrogen dioxide. ^a^ Adjusted for age, BMI, tobacco use, physical activity, education level, fruit and vegetable intake, alcohol use, type of fuel used home, household annual income, and location of residence (urban/rural). ^b^ Adjusted for age, sex, BMI, physical activity, education level, fruit and vegetable intake, alcohol use, type of fuel used at home, household annual income, and location of residence (urban/rural). ^c^ Adjusted for sex, BMI, tobacco use, physical activity, education level, fruit and vegetable intake, alcohol use, type of fuel used at home, household annual income, and location of residence (urban/rural). ^d^ Adjusted for age, sex, BMI, tobacco use, physical activity, education level, fruit and vegetable intake, alcohol use, type of fuel used at home, and location of residence (urban/rural).

## Data Availability

Data may be obtained from a third party and are not publicly available. We used data from the WHO Study on global AGEing and adult health (SAGE) to analyze and report the findings. Data access policy is available on https://apps.who.int/healthinfo/systems/surveydata/index.php/catalog/13 (accessed on 24 October 2013).

## Supplementary Materials

The following are available online at https://www.mdpi.com/1660-4601/18/6/3258/s1: Figure S1. Percentage change (95% CI) in hs-CRP levels associated with 10 ug/m^3^ increase in 5 year moving averages of air pollution; Figure S2. Percentage change (95% CI) in hs-CRP levels associated with 10 μg/m^3^ increase in 1 year moving averages of air pollution; Table S1. Results of 10-fold cross-validation for PM_1_, PM_2.5_, PM_10_, and NO_2_; Table S2. Characteristics of the study participants and nonparticipants (without CRP sample).
