# Supplementary material for: Systemic Inflammation (C-Reactive Protein) in Older Chinese Adults Is Associated with Long-Term Exposure to Ambient Air Pollution
Source: Int J Environ Res Public Health. 2021 Mar 22;18(6):3258. doi: 10.3390/ijerph18063258 (PMC8004276; doi:10.3390/ijerph18063258)
Supplement: Supplementary file 1 [file ijerph-18-03258-s001.pdf]

Figure S1: Percentage change (95% CI) in hs-CRP levels associated with 10 ug/m^3 increase in 5-years moving averages of air pollution.

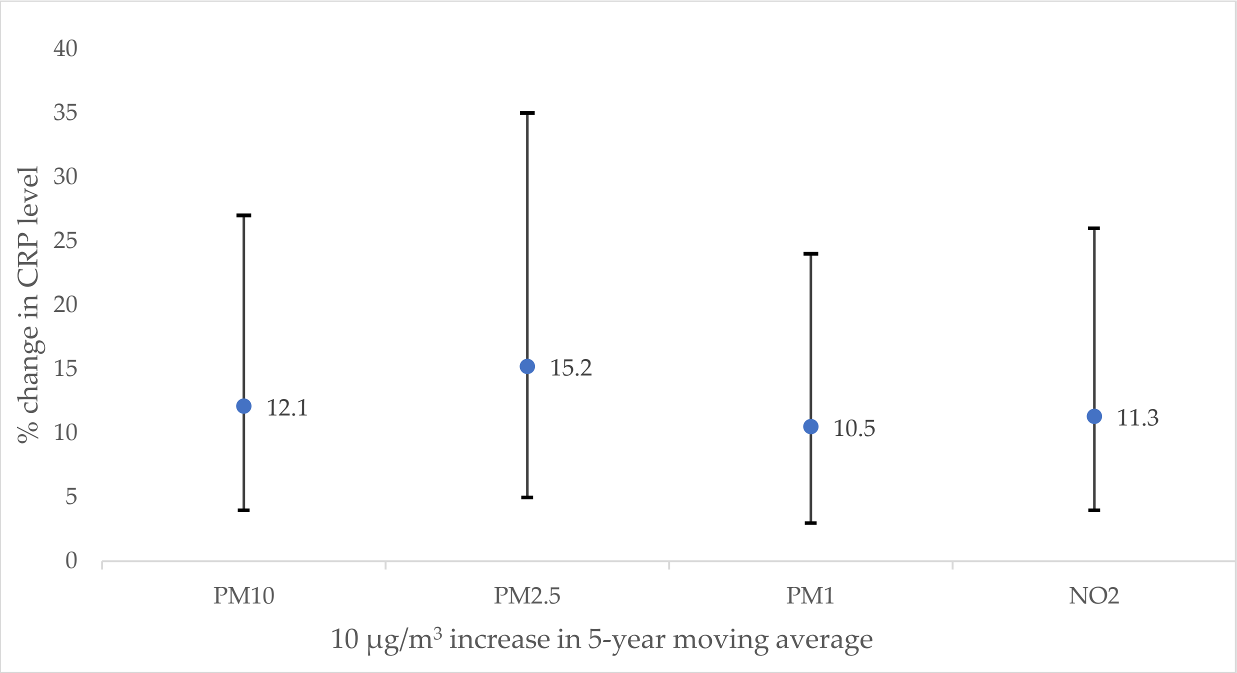

PM_{10}, particulate matter with a diameter of 10 µm or less; $PM_{2.5}$, with a diameter of 2.5 µm or less; PM_1, particulate matter with a diameter of 1 µm or less; NO_2, nitrogen dioxide.

Figure S2: Percentage change (95% CI) in hs-CRP levels associated with 10 μg/m^3 increase in 1-year moving averages of air pollution.

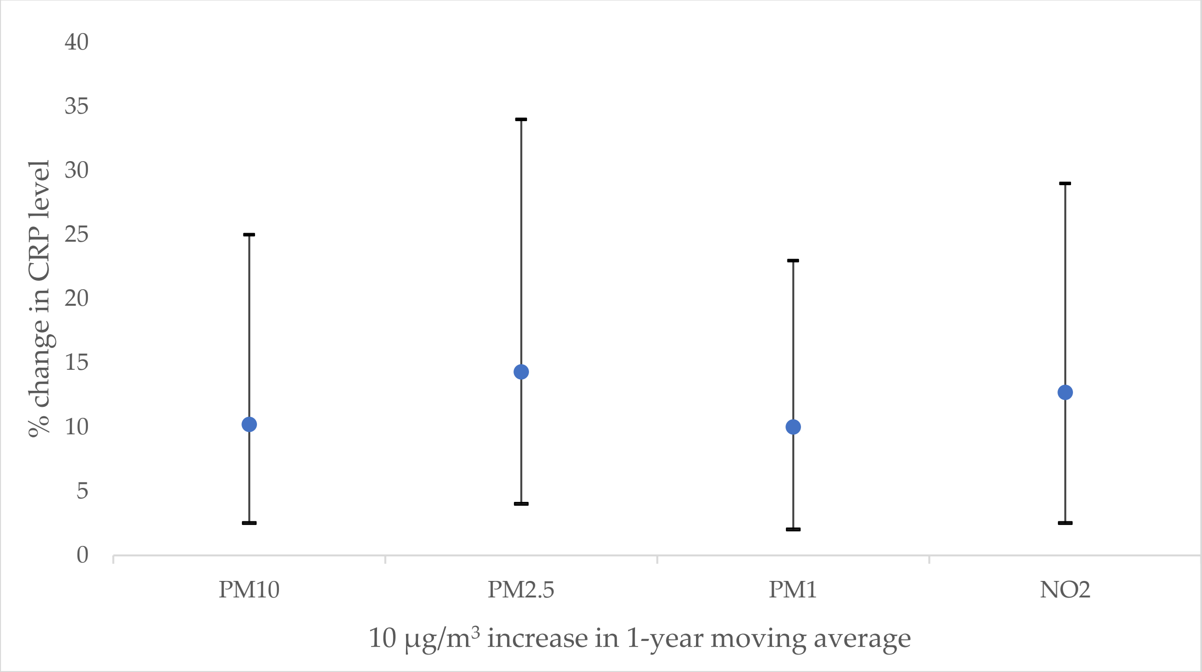

PM_{10}, particulate matter with a diameter of 10 μm or less; $PM_{2.5}$, with a diameter of 2.5 μm or less, PM_1, particulate matter with a diameter of 1 μm or less; NO_2, nitrogen dioxide.

Table S1. Results of 10-fold cross-validation for PM_1, $PM_{2.5}$, PM_{10}, and NO_2.

**Pollutants**	**Daily Model**		**Annual Averages**	
	**CV R^2**	**RMSE**	**CV R^2**	**RMSE**
PM_1	55%	20.5 μg/m^3	75%	8.8 μg/m^3
$PM_{2.5}$	83%	18.1 μg/m^3	86%	6.9 μg/m^3
PM_{10}	78%	31.5 μg/m^3	81%	14.4 μg/m^3
NO_2	64%	12.4 μg/m^3	72%	6.5 μg/m^3

RMSE: Root mean square error; CV: cross-validation.

Table S2. Characteristics of the study participants and non-participants (without CRP sample).

	**No (n)**	**%**	**Yes (n)**	**%**	**p-value***
**CRP**	5,452	40.8%	7,915	59.2%	
**Age (mean, SD)**	63.1 (9.6)		63.2 (9.4)		0.392
**Sex**					
Female	2,952	41.6%	4,141	58.4%	0.000
Male	2,500	39.8%	3,774	60.2%	
**Smoking**					
Ever smoker	3,368	39.0%	5,275	61.0%	0.000
Never smoker	1,707	39.5%	2,615	60.5%	
**Alcohol**					
No	3,458	38.6%	5,496	61.4%	0.561
Yes	1,614	40.4%	2,377	59.6%	
**BMI (mean, SD)**	23.9 (5.2)		24.1 (4.8)		0.006
**Physical activity**					
Low level	1,475	35.3%	2,707	64.7%	0.000
Moderate	1,402	38.5%	2,243	61.5%	
High	2,198	42.8%	2,939	57.2%	
**Education**					
No school	2,399	42.8%	3,201	57.2%	0.000
Primary	956	36.7%	1,646	63.3%	
Middle	1,173	43.3%	1,535	56.7%	
Higher education	904	37.9%	1,481	62.1%	
**Fruit and vegetable consumption**					
Insufficient	2,704	44.8%	3,336	55.2%	0.000
Sufficient	2,748	37.5%	4,579	62.5%	
**Fuel used at home**					
Clean	2,790	37.8%	4,588	62.2%	0.000
Unclean	2,650	44.8%	3,265	55.2%	
**Annual household income**					
≤15,000¥	3,147	47.1%	3,529	52.9%	0.000
>15,000¥	2,263	35.4%	4,129	64.6%	
**Location of residence**					
Rural	2,524	37.1%	4,276	62.9%	0.000
Urban	2,928	44.6%	3,639	55.4%	
